# Lymphedema after breast cancer

**DOI:** 10.11604/pamj.2016.23.255.9585

**Published:** 2016-04-28

**Authors:** Sami Aziz Brahmi, Fatima Zahra Ziani

**Affiliations:** 1 Service d'Oncologie Médicale, Centre Hospitalier Mohammed VI, Oujda, Maroc; 2 Service d'Oncologie Médicale, Centre Hospitalier, Hassan II, FES, Maroc

**Keywords:** Lymphedema, breast cancer, complication

## Image in medicine

Lymphedema is one of the most significant survivorship issues after the surgical treatment of breast cancer and in this population it has been documented to have significant quality of life consequences. It is the result of obstruction or disruption of the lymphatic system, which can occur as a consequence of tumors, surgery, trauma, and radiation therapy, and this is lead to the accumulation of fluid in the interstitial tissues. We report the case of a patient referred in our department after breast surgery (Patey intervention) for adjuvant chemotherapy. The patient received adjuvant chemotherapy and adjuvant radiotherapy. She developed one year after completion of the treatment a swelling in the right arm, and hand, with a temporary indentation of the skin after finger pression (pitting edema). An ultra sound exam was performed and deep vein thrombosis was excluded. The patient was referred to a physical therapist for management of her lymphedema. Cancer related lymphedema is a common post treatment complication. In breast cancer patients, lymphedema has been described as an often underdiagnosed, and undertreated condition. Also, early detection of lymphedema increases the likelihood of successful treatment. Patients must be informed and conservative surgery like sentinel lymph nodes technique should be performed to reduce the risk.

**Figure 1:**
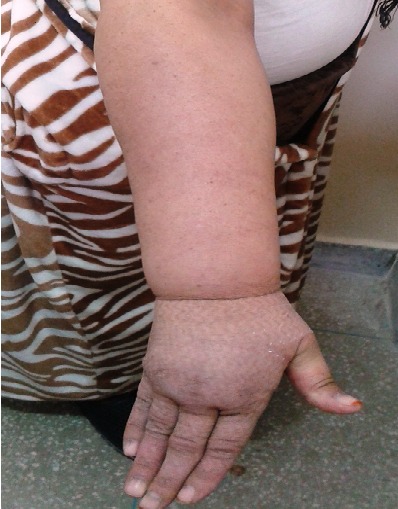
Lymphedema of the right arm and hand

